# Glucocorticoid signaling in the liver and adipose tissue of male and female fructose-fed rats

**DOI:** 10.1186/1753-6561-6-S3-P35

**Published:** 2012-06-01

**Authors:** Gordana Matić, Nataša Veličković, Ana Djordjevic, Danijela Vojnović Milutinović, Ivana Elaković, Jelena Nestorov, Biljana Bursać, Ana Vasiljević, Marina Nikolić, Jadranka Dundjerski

**Affiliations:** 1 Institute for Biological Research “Siniša Stanković”, University of Belgrade, 11060 Belgrade, Serbia

## Background

The rise in consumption of refined sugars high in fructose appears to be an important factor contributing to epidemic of obesity and metabolic syndrome [1]. Fructose is involved in the genesis and progression of the syndrome through deregulation of metabolic pathways in the liver and adipose tissue, as sites of insulin-modulated metabolism [2]. Enhanced regeneration of glucocorticoids within the liver and adipose tissue, mediated by the enzyme 11beta-hydroxysteroid dehydrogenase type 1 (11βHSD1), may contribute to adiposity and metabolic disease [3]. 11βHSD1 reductase activity is crucially dependent on NADPH, a cofactor generated by the enzyme hexose-6-phosphate dehydrogenase (H6PDH) [4]. We hypothesized that harmful effects of high fructose consumption are mediated by alterations in prereceptor metabolism of glucocorticoids and in the level of glucocorticoid receptor (GR) expression and compartmental redistribution in the liver and adipose tissue. We also assume that high fructose intake differently affects glucocorticoid signaling in the liver and adipose tissue of male and female rats.

## Materials and methods

Symptoms of metabolic syndrome in 12-week old male and female Wistar rats were analyzed after 9-week intake of 10% fructose solution instead of water. Protein and mRNA levels of 11βHSD1, H6PDH and GR were determined in the liver and adipose tissue by Western blot and qPCR analyses, respectively. Plasma and tissue corticosterone levels were measured by ELISA.

## Results

In male rats, insulin sensitivity was impaired, while blood pressure, non-esterified fatty acid (NEFA) release and plasma triglycerides level were increased by fructose diet. In the adipose tissue, the fructose-provoked increase in corticosterone level was accompanied by enhanced 11βHSD1 and H6PDH expression, as well as by stimulated GR translocation to the nucleus. In the liver, high fructose diet led to elevation of 11βHSD1 protein and GR nuclear accumulation, while H6PDH mRNA and corticosterone level were not changed. In fructose fed females, mass of visceral adipose tissue and plasma triglycerides level were increased, while blood pressure and insulin level and sensitivity were unaffected by fructose intake. The rise of corticosterone in the adipose tissue was accompanied by GR protein decline in both the cytoplasm and nuclei.

## Conclusions

The results demonstrate that fructose-related elevation of triglycerides and NEFA in blood plasma of male rats coincides with enhanced prereceptor glucocorticoid metabolism in the adipose tissue. The observed gender-specific differences in metabolic phenotype might derive from differences in GR expression and intracellular redistribution in the adipose tissue.
